# Pore-scale mass transfer heterogeneity shapes nutrient accessibility and functional assembly in porous microbial ecosystems

**DOI:** 10.1093/ismejo/wraf205

**Published:** 2025-09-12

**Authors:** Liming Wu, Daixiu Bao, Hui Liao, Meiyu Yan, Yitong Ge, Zinuan Han, Xiaole Xia

**Affiliations:** College of Food Science and Engineering, Tianjin University of Science and Technology, Tianjin, 300457, P. R. China; College of Food Science and Engineering, Tianjin University of Science and Technology, Tianjin, 300457, P. R. China; The Key Laboratory of Industrial Biotechnology, Ministry of Education, School of Biotechnology, Jiangnan University, Wuxi, 214122, P. R. China; College of Food Science and Engineering, Tianjin University of Science and Technology, Tianjin, 300457, P. R. China; College of Food Science and Engineering, Tianjin University of Science and Technology, Tianjin, 300457, P. R. China; College of Food Science and Engineering, Tianjin University of Science and Technology, Tianjin, 300457, P. R. China; College of Food Science and Engineering, Tianjin University of Science and Technology, Tianjin, 300457, P. R. China; The Key Laboratory of Industrial Biotechnology, Ministry of Education, School of Biotechnology, Jiangnan University, Wuxi, 214122, P. R. China

**Keywords:** porous ecosystems, heterogeneity, substrate degradation, community assembly, microfluidics

## Abstract

Porous ecosystems represent ubiquitous microbial habitats across natural settings including soil, gut tract, and food matrices, where microscale spatial architecture critically shapes microbial colonization and interactions. Yet, the mechanisms of how pore-scale physical constraints influence microbial community assembly and metabolic performance remain poorly understood. Here, we employed a microfluidic platform with tunable inter-pillar spacings, coupled with a multi-omics approach including *in situ* imaging, exometabolomics, metagenomics, and metatranscriptomics, to investigate how pore-size modulates microbial community dynamics. Comparing representative small (50 μm) and large (150 μm) pore-sizes, we found that larger pore-sizes promoted greater biomass accumulation and significantly enhanced exometabolite production, particularly of amino acids. Microscopy and quantitative assays revealed that 150 μm pores facilitated more efficient substrate degradation, especially of carbohydrates. Taxonomic profiling showed that increasing pore-size reduced community evenness while enhancing richness, selectively enriching carbohydrate-degrading and amino acid-producing taxa, and promoting more complex, positively correlated co-occurrence networks. Metatranscriptomic analysis further demonstrated that larger pore-size significantly upregulated key functional genes involved in substrate degradation, amino acid biosynthesis, and stress response pathways. Fluorescent tracer assays revealed pronounced mass transfer heterogeneity, where smaller pores exhibited prolonged solute persistence and steeper chemical gradients, ultimately restricting substrate availability and microbial activity. Collectively, our results reveal that alleviation of microscale spatial constraints enhances nutrient accessibility, metabolic function, and community organization in porous ecosystems, underscoring the pivotal role of physical microstructure in regulating both the taxonomic composition and functional capacity of microbial ecosystems.

## Graphical Abstract

**Graphical Abstract:**
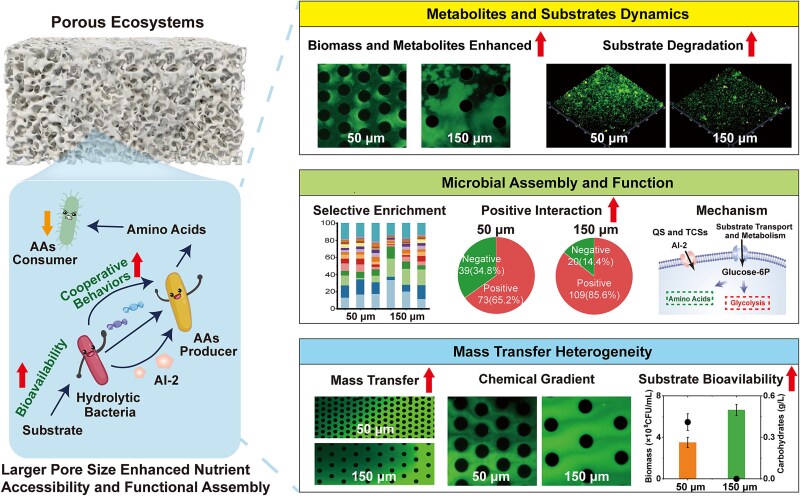

## Introduction

Bacteria predominantly inhabit structurally complex porous environments, including soils [1, 2], gastrointestinal tracts [3], and fermented food matrices [4]. These habitats are characterized by interconnected three-dimensional pore networks that give rise to spatially heterogeneous microenvironments, which strongly influence microbial colonization, spatial organization, and interspecies metabolic interactions [5–7]. In turn, microbial activity profoundly alters the physical and chemical properties of these environments, driving key processes such as biogeochemical cycling and bioremediation [8, 9]. Therefore, understanding how microbial communities assemble and function within these physically structured environments is critical for advancing microbial ecology and informing microbiome-based strategies in environmental, industrial, and clinical settings.

Microscale pore architecture, including size distribution, tortuosity, and connectivity, is widely recognized as a key determinant of microbial community structure and ecosystem function [10, 11]. Pore-sizes can span several orders of magnitude, from millimeter-scale channels to nanometer-scale constrictions [12]. In soils, e.g. sand-associated pores range from 50 to 2000 μm, silt-associated pores from 2 to 50 μm, and clay-associated pores below 2 μm [13]. Pore-sizes around 50 μm and 150 μm are widely recognized as representative scales in natural ecosystems, reflecting the fundamental heterogeneity of pore space common across diverse habitats [14]. This multiscale physical heterogeneity gives rise to spatially distinct microniches that influence microbial localization, metabolic exchange, and ecological interaction networks [15]. Empirical studies have shown that large soil pores promote faster carbon turnover and select for specific microbial taxa compared to smaller pores using x-ray computed microtomography [16]. Similarly, in solid-state fermentation systems, porosity has been linked to the efficiency of amino acid production and microbial succession [17]. Despite these insights, direct evidence of how pore-scale physical heterogeneity structures microbial communities remains limited due to the opaque nature of particulate media and challenges associated with *in situ* visualization.

Microfluidic platforms provide a versatile and high-resolution approach to investigate microbial behavior and interactions in porous environments [13, 18, 19]. By mimicking porous networks using arrays of micropillars with tunable inter-pillar spacings, these systems enable precise control of physical architecture while allowing real-time, *in situ* visualization via advanced microscopy [3, 20]. Recent studies have leveraged these capabilities to uncover key insights into microbial colonization dynamics, biofilm development, cell motility, and metabolite exchange [6, 21, 22]. However, the majority of such studies focus on simplified consortia composed of a few strains such as *Escherichia coli* [3] and *Bacillus subtilis* [23, 24], thereby overlooking the diversity and complexity of microbial interactions in natural communities.

Beyond physical structure, mass transport dynamics in porous media are also profoundly shaped by spatial heterogeneity [25–27]. Irregularities in pore geometry and size give rise to complex velocity fields, which influence advective and diffusive transport of solutes [11, 28, 29]. Additionally, natural porous systems often exhibit chemical heterogeneity due to localized nutrient sources, such as carbon-rich aggregates in soils [16, 20]. These sources give rise to heterogeneous concentration fields and steep microscale chemical gradients that influence microbial behaviors, including chemotaxis, aggregation, and metabolic specialization [30, 31]. Experimental and modeling studies have suggested that the interplay between physical structure, mass transfer, and microbial traits—such as motility and quorum sensing (QS)-governs spatially structured microbial activity [3, 32]. However, most studies often neglect the multiscale heterogeneity of pore structure and the diversity of microbial communities, leading to an incomplete understanding of how pore-scale constraints jointly affect community assembly, function, and substrate utilization in diverse microbial consortia.

To address knowledge gaps, we investigated how pore-size-driven spatial constraints regulate microbial community assembly, metabolic function, and substrate degradation. Using microfluidic platforms with precisely defined pore-sizes (50 μm and 150 μm), we examined how different pore scales affect mass transfer, microbial colonization, and metabolic outputs. We applied a multi-omics, including exometabolomics, metagenomics, and metatranscriptomics, to characterize taxonomic composition, functional gene expression, and metabolite profiles across pore conditions. We further employed fluorescent tracer assays and COMSOL modeling to characterize mass transfer persistence and chemical gradient formation to evaluate substrate availability. Our study reveals that pore-size driven mass transfer heterogeneity is a critical physical determinant that governs microbial spatial organization, modulates metabolic capacity, and shapes community structure, with these physically constrained principles being directly relevant to other porous microbial ecosystems including soils and host-associated environments such as the gastrointestinal tract.

## Materials and methods

### Microfluidic devices and experiments

Microfluidic chambers were engineered to mimic porous environmental architectures using a matrix of micropillars (50 μm diameter and height). Two distinct microfluidic designs were fabricated, differing in interpillar spacing to produce pore-sizes of 50 μm and 150 μm, respectively. Natural porous fermentation system *Daqu* samples were collected in March 2024 from a traditional solid-state fermentation storeroom in Jiangsu, China (35°52′51.50″N, 119°46′12.87″E). Microbial cells were isolated from the samples using Nycodenz density gradient centrifugation. Isolated microbial cells were initially precultivated in the extract medium to promote community acclimation, then harvested and resuspended in phosphate-buffered saline to an optical density at 600 nm of 0.1. Subsequently, 100 μl of the bacterial suspension was introduced into sterilized microfluidic chips. Following a 1-h static attachment phase, a continuous perfusion of sterile extract medium was applied at a flow rate of 0.1 μl/min using a syringe pump. Microbial community development was monitored over a 3 days period at 37°C, a temperature that supports the growth and metabolic activity of core functional microbial populations within the *Daqu* microbiome, to quantify biomass accumulation and assess spatial colonization dynamics. The *Daqu* extract medium was prepared from the same *Daqu* sample using a modified method [20]. Negative controls using uninoculated microfluidic chips containing only the culture medium were included in parallel for each experimental condition. The details of preparation methods are described in the supplementary information.

### Microscopy and image processing

Microbial cells within the microfluidic chambers were stained with the nucleic acid-binding dye SYTO 9 (Thermo Fisher Scientific) for 30 min in the dark at room temperature [20]. Fluorescent imaging was performed using a confocal laser scanning microscope (CLSM; LSM900, Carl Zeiss, NY, United States). To assess substrate composition, carbohydrates were stained with FITC-ConA and proteins were labeled with SYPRO Orange (Sigma-Aldrich) [33]. To evaluate morphological changes in the substrate associated with different pore architectures, samples from the 50 μm and 150 μm microfluidic systems were fixed and imaged using scanning electron microscopy (SEM; SU1510, Hitachi, Tokyo, Japan). Fluorescence images were analyzed using ImageJ (NIH, Bethesda, MD), with quantitative colocalization analyses based on mean fluorescence intensity.

### Exometabolomic analysis

To assess the influence of pore-size on microbial metabolic activity, 100 μl of effluent at d 3 was collected from the microfluidic chip outlet. Metabolomic profiling was performed using an ultra-high performance liquid chromatography system (Vanquish UHPLC) coupled to a Q Exactiv HF-X hybrid quadrupole-Orbitrap mass spectrometer (Thermo Fisher Scientific). Data acquisition was carried out in both positive and negative electrospray ionization (ESI) modes, with a full-scan mass range of m/z 100–1500. The raw data were converted to mzXML format using ProteoWizard, and subsequently processed with XCMS for peak detection and alignment. Metabolite identification was performed based on accurate mass and MS/MS spectral matching against reference libraries, including HMDB, LipidMaps, and mzCloud. The XCMS parameters were set as follows: method = “centWave”, ppm = 10, peakwidth = c (10, 60), mzwid = 0.025, minfrac = 0.5, and bw = 5. Only metabolites with level 2 identification (putatively annotated compounds) were retained for downstream statistical analysis. The details of analytic methods are described in the supplementary information.

### Substances concentrations quantification

For each quantification analysis, 100 μl of effluent collected on Days 1, 2, and 3 was filtered through a 0.2 μm membrane to remove residual cells. The total concentration of free amino acids was quantified using an amino acid assay kit (Solarbio, Beijing, China). Amino acid composition was determined using an automated amino acid analyzer (L-8900, Hitachi Ltd., Tokyo, Japan). Soluble carbohydrates were quantified using the anthrone-sulfuric acid method, while soluble protein concentrations were determined with a BCA protein assay kit (Boxbio, China) according to the manufacturer's instructions. In parallel, the chemical composition of substrate groups was characterized using Fourier-transform infrared spectroscopy (FTIR; Nicolet 6700, Thermo Fisher Scientific, United States).

### Metagenomic and metatranscriptomic analysis

To investigate microbial community composition across different pore-size conditions, resident microbial cells were harvested from the microfluidic chambers at Day 3. Genomic DNA was extracted using the E.Z.N.A. Soil DNA Kit (Omega Bio-Tek, United States) following the manufacturer's protocol. DNA concentration was assessed using a Thermo NanoDrop One (Thermo Fisher Scientific, MA, United States). DNA was then fragmented to an average size of ~400 bp using a Covaris M220 ultrasonicator (Gene Company Limited, China) to prepare for paired-end library construction. Libraries were prepared using the NEXTFLEX Rapid DNA-Seq Kit (Bioo Scientific, Austin, TX, United States), during which adapters containing the full complement of sequencing primer hybridization sites were ligated to the blunt-ended fragments. Paired-end sequencing was performed on a NovaSeq 6000 System using the S4 Reagent Kit v1.5 (2 × 150 bp, 300 cycles, insert size ~350 bp). On average, each sample yielded ~13 gb of high-quality sequencing data. Raw reads were processed with fastp to remove adapter sequences and filter out low-quality reads (length < 50 bp, average quality score < 20, or containing ambiguous bases). Clean reads were assembled de novo using MEGAHIT, which employs succinct de Bruijn graph algorithms for efficient metagenomic assembly. Contigs with lengths ≥300 bp were retained as the final assembly (N50 ~453 bp; N90 ~323 bp) and subsequently used for downstream gene prediction and functional annotation. A nonredundant gene catalog was constructed using CD-HIT (http://www.bioinformatics.org/cd-hit/) with 90% sequence identity and 90% coverage. High-quality reads were aligned to the nonredundant gene catalogs to calculate gene abundance with 95% identity using SOAPaligner (http://soap.genomics.org.cn/, version 2.21). Representative sequences of nonredundant gene catalog were aligned to NR database with an e-value cutoff of 1e-5 using Diamond (http://www.diamondsearch.org/index.php) for taxonomic annotations. Bioinformatics analyses, including community composition profiling, alpha and beta diversity, taxonomic comparison and co-occurrence networks were conducted using the Majorbio Cloud Platform (http://www.majorbio.com).

Total RNA was extracted using the TRIzol Reagent (Invitrogen, MA, United States) following the manufacturer’s protocol. RNA concentration and purity were assessed using a NanoDrop 2000 spectrophotometer (Thermo Fisher Scientific, MA, United States), and RNA integrity was verified by agarose gel electrophoresis. mRNA sequencing libraries were prepared using the TruSeq Stranded mRNA Library Prep Kit (Illumina, MA, United States). Sequencing was performed on a NovaSeq 6000 System (2 × 150 bp, 300 cycles, insert size ~300 bp), generating ~16 gb of paired-end reads per sample (N50 ~1030 bp; N90 ~344 bp). To ensure accurate comparisons of gene expression across different genes, transcript levels were normalized using transcripts per million (TPM). Relative gene activity was similarly evaluated based on normalized TPM values, enabling functional comparisons across samples. Moreover, the representative genes were detected by qRT-PCR analysis using the primers listed in Supplementary Table S1.

### Strain isolation and evaluation of metabolic activity

Enriched bacterial strains were isolated from the effluent of porous chambers using serial dilution and plating on LB agar. Isolates were identified by 16S rRNA gene sequencing, and one dominant strain was classified as *B. subtilis* Bs01. To assess its metabolic potential under different pore-size conditions, Bs01 was inoculated into microfluidic chips, and biomass was quantified by colony-forming units (CFUs). A 100 μl aliquot of effluent samples were analyzed for substrate utilization and amino acid content using the aforementioned methods. Furthermore, to evaluate hydrolytic activity, a reporter strain was constructed by introducing *egfp* under the control of the *amyE* promoter using the primers listed in Supplementary Table S1. Amylase activity was assessed via fluorescence intensity and confirmed using an amylase assay kit (Solarbio, Beijing, China).

### Statistics analysis

All of experiments were performed at least three times. Results are presented as mean ± standard deviation (SD). Statistical significance was evaluated using an unpaired two-tailed Student’s t-test to determine the *P*-value. ^*^P < .05; ^**^P < .01; ^***^P < .001.

## Results and discussion

Microfluidic investigation of pore-size effects on bacterial biomass and metabolism in porous ecosystems.

**Figure 1:**
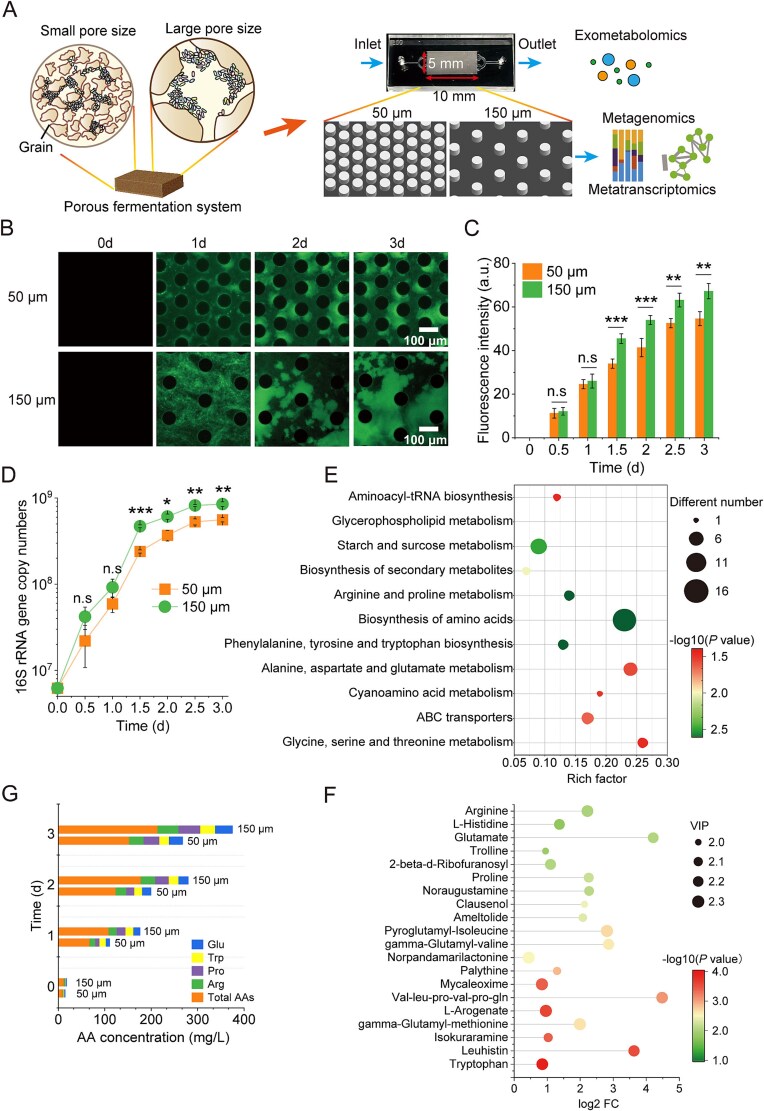
A microfluidic platform for studying the effect of pore-size on microbial community assembly and function. (A). The microfluidic chips contain micropillars to simulate 50 μm and 150 μm pore-sizes for microbial community development. (B). Time-lapse imaging of community architecture development in the porous environment. Cells were stained with SYTO 9 (green). The assay was conducted in five independent replicates. (C). Fluorescence intensity of microbial communities in 50 μm and 150 μm pore-size chips (*n* = 5) over 3 days of incubation. (E). KEGG pathway enrichment analysis of differential metabolites between 50 μm and 150 μm pore-sizes. Enrichment significance was calculated using Fisher’s exact test, and *P*-values were adjusted for multiple comparisons using the false discovery rate (FDR) correction. Metabolomics was performed in three replicates. (F). Top 20 differentially expressed metabolites (by fold change) between 50 μm and 150 μm pore-sizes. (G). Amino acid concentrations in 50 μm and 150 μm pore-size systems during 3-day incubation, measured by amino acid analyzer (*n* = 5). Total amino acids represent the sum of all other detected amino acids, including Glu, Trp, Pro, and Arg, which are shown individually. Data are presented as mean ± SD.

To investigate how pore-scale spatial constraints influence microbial community assembly and metabolism, we developed two innovative PDMS-based microfluidic platforms that mimic the structural heterogeneity of natural porous environments. Each device featured regularly spaced pillar arrays with defined interpillar distances of 50 μm or 150 μm, which are widely recognized as representative scales in natural ecosystems (Fig. 1A). The devices were inoculated with a natural microbial community from the *Daqu* porous fermentation system-a highly porous solid food matrix produced through spontaneous, open fermentation that harbors diverse environmental microorganisms [34] and cultivated using an extract medium derived from the same source. In the 50 μm pore-size, microbial colonization initiated with the formation of discrete microcolonies anchored to the pillar surfaces. Within 2 days, these microcolonies expanded, bridging adjacent pillars, infiltrating pore spaces, and ultimately leading to near-complete channel occlusion (Supplementary Fig. S1). In contrast, in the 150 μm pores, cells predominantly proliferated as planktonic aggregates within the pore voids, exhibiting reduced pillar attachment and more dispersed spatial organization (Fig. 1B; Supplementary Fig. S2). Time-lapse imaging over 3 days confirmed that both systems reached stationary phase by Day 3 (Supplementary Fig. S3). Quantitative fluorescence imaging revealed that the 150 μm pore system exhibited significantly higher biomass-associated fluorescence intensity than the 50 μm system (*P* < .001 at Day 2; *P* < .01 at Day 3; Fig. 1C). These findings were corroborated by qPCR quantification of 16S rRNA gene copy numbers, which indicated higher bacterial DNA levels in the larger-pore environment except at the 1 day incubation (Fig. 1D).

To assess how pore-size influenced community-level exometabolism, we conducted untargeted metabolomics analysis of microfluidic chip effluents collected at 3 days of fermentation. We detected 1417 and 1542 metabolites in the 50 μm and 150 μm systems, respectively, with fatty acids and amino acids (and their derivatives) constituting major constituents (Supplementary Fig. S4). Enrichment analysis revealed that the 150 μm pore-size significantly upregulated key metabolic pathways, particularly those involved in amino acid biosynthesis, including aromatic amino acids, arginine (Arg) and proline (Pro) metabolism, and glutamate (Glu) family pathways (Fig. 1E). Orthogonal partial least squares-discriminant analysis (OPLS-DA) also showed robust separation between two porosities, with 109 discriminant features (VIP > 1.0) (Supplementary Fig. S5). Amino acid-related metabolites constituted 41.2% of the upregulated compounds (Fig. 1F; Supplementary Fig. S6). Meanwhile, the temporal changes in amino acids concentrations were determined by an amino acid analyzer. The total amino acid concentration in the 150 μm condition was 1.4-fold higher than that in the 50 μm condition. The differential accumulation was primarily driven by four key amino acids Arg, Pro, tryptophan (Trp), and Glu, which accounted for 68.4% of the observed concentration differences between pore-sizes (Fig. 1G). Our results revealed that larger pore-sizes (150 μm) significantly enhanced biomass accumulation and exometabolite production, particularly amino acids (*P* < .01). Amino acids are not only essential for microbial growth but also act as key signaling molecules, osmoprotectants, and intermediates in central metabolic pathways [35]. In the gut microbiome, inter-bacterial mutualistic interactions can be facilitated by amino acids shared as public goods [36]. Similarly, in solid-state fermentation systems, variations in porosity have been shown to affect amino acid production, making them a useful functional marker for evaluating fermentation performance [17, 37].

Larger pore-size facilitates substrate degradation and enhances metabolite production.

**Figure 2:**
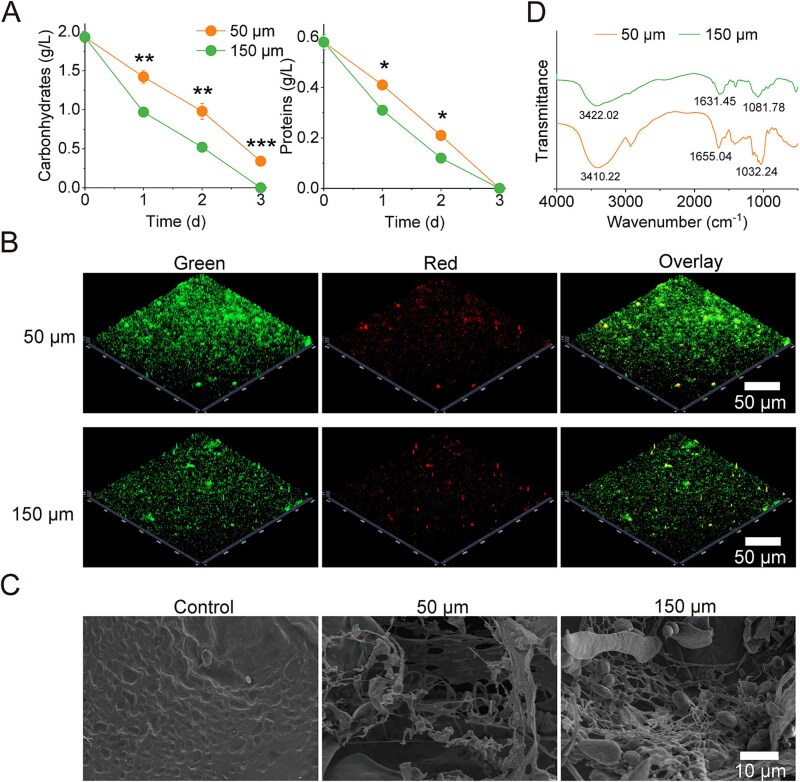
Substrate degradation was promoted in the larger pore-size system. (A). Carbohydrate and protein contents in the 50 μm and 150 μm pore-size systems during the 3-day incubation period (*n* = 3). (B). Representative CLSM images at Day 2 (green: carbohydrates; red: proteins). *C. SEM* Images of substrate degradation in the 50 μm and 150 μm pore-size systems at Day 2. D. FTIR spectra of spent substrates in the 50 μm and 150 μm pore-size systems at Day 2. Data are presented as mean ± SD. ^*^P < .05. ^**^P < .01.

Building upon previous research demonstrating the critical role of substrate degradation in determining metabolic productivity during porous ecosystems [33, 38], we quantitatively characterized the degradation dynamics of primary nutrient substrates soluble proteins and carbohydrates across different pore-size conditions. The initial medium contained mainly carbohydrates (1.93 ± 0.07 g/l) and soluble proteins (0.58 ± 0.03 g/l), corresponding to 2.83 g C/kg total organic carbon, which served as the baseline for assessing degradation efficiency. The 150 μm pore-size demonstrated superior degradation performance, with carbohydrate content decreasing by 73.1% at Day 2, significantly faster than the 49.2% reduction observed in the 50 μm (*P* < .01). Similarly, soluble protein degradation rates showed pore-size dependence (*P* < .05) (Fig. 2A). CLSM imaging with fluorescent probes for polysaccharides and proteins revealed markedly lower fluorescence intensities in the 150 μm pores, indicating more extensive substrate degradation (Supplementary Fig. S7; Fig. 2B).

SEM observations further corroborated these findings at the microstructural level, showing that while the original substrate exhibited a compact, homogeneous morphology, substrates in 50 μm produced a fluffy, loosely-organized structure, and 150 μm generate more fragmented architectures with extensive surface area exposure (Fig. 2C). FTIR analysis of spent substrates revealed pore-size dependent changes in characteristic absorption bands: the 150 μm showed greater reduction in corresponding to the C-N amide I group of protein (1650 cm^−1^) and carbohydrate-region signals (900–1200 cm^−1^) compared to 50 μm, confirming more extensive breakdown of both proteinaceous and carbohydrate components in the larger-pore system. (Fig. 2D). Overall, the above results implied that the 150 μm facilitated the substrates degradation, as evidenced by the pronounced reduction in protein and particularly carbohydrate content, which contributes to increased metabolites production. These findings are consistent with previous studies reporting that pore space can modulate substrate turnover [39]. For example, larger pores tend to promote rapid influx and subsequent decomposition of newly introduced carbon in soil aggregates, whereas lower carbon losses have been associated with smaller pores in the 6.5–40 μm ranges [40, 41].

### Selective enrichment of functional microbial taxa within larger pore-sizes

**Figure 3:**
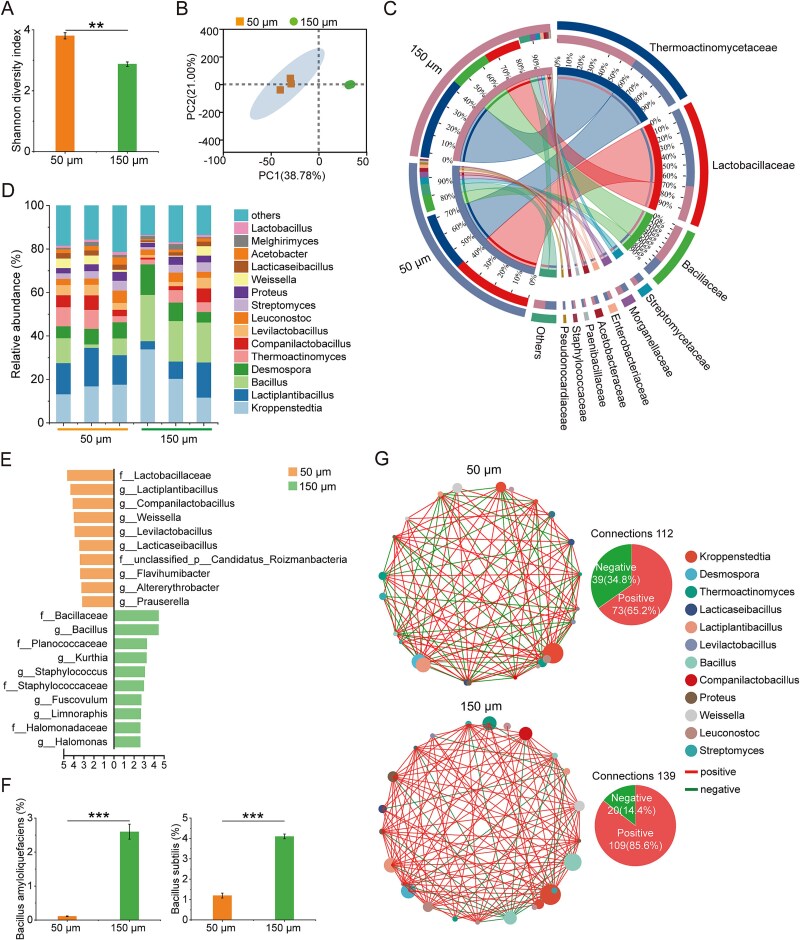
Preferential enrichment of functional microorganisms in larger pore environments. (A). Shannon diversity of microbial communities in 50 μm and 150 μm pore-sizes. (B). PCA of microbial communities in the 50 μm and 150 μm groups. (C). Relative microbial abundance at the family level. (D). Relative microbial abundance at the genus level. (E). LDA scores of differentially expressed genera between the 50 μm and 150 μm groups. (F). Relative abundances of *B. Amyloliquefaciens* and *B. Subtilis*, which were significantly higher in the 150 μm pore-size group. (G). Co-occurrence networks of the first 25 species were constructed using Spearman’s rank correlation, highlighting both positive and negative correlations (Spearman’s r > 0.5, *P* < .05). Metagenomics was performed in three replicates. Data are presented as mean ± SD. ^**^P < .01; ^***^P < .001.

Biochemical transformations within complex porous ecosystems are intrinsically shaped by the composition and spatial organization of functional microbial consortia [6, 42]. Our high-resolution metagenomic profiling revealed significant pore-size dependent reorganization of microbial populations, with distinct taxonomic and functional differentiation between the 50 μm and 150 μm pore environments. Quantitative α-diversity analysis revealed pronounced effects of pore architecture, with a significant decrease in the Shannon index from the 50 μm to the 150 μm system (Fig. 3A), reflecting reduced community evenness but increased taxonomic richness in the larger-pore environment (Supplementary Fig. S8). Principal component analysis (PCA) further revealed a clear separation of the 150 μm from the 50 μm group (Fig. 3B). Taxonomic analysis revealed both systems were dominated by members of *Thermoactinomycetaceae*, *Lactobacillaceae*, and *Bacillaceae* (Fig. 3C and D). However, the 150 μm pore-size selectively enriched for taxa with known hydrolytic and biosynthetic capabilities. Specifically, *Thermoactinomycetaceae* and *Bacteroidota* were more abundant in the 150 μm system whereas *Lactobacillaceae* abundance was reduced (Fig. 3C, Supplementary Table S2). Linear discriminant analysis effect size (LEfSe) bar plots further highlighted the genera that best represented the largest differences between the two pore-sizes (LDA score > 2.5) (Fig. 3E). Hydrolytic and amino acid-producing genera, such as *Kroppenstedtia* and *Bacillus*, were significantly enriched in the 150 μm environment. In contrast, potential amino acids consumers, e.g. *Lactiplantibacillus*, exhibited a significant downward trend in abundance in the 150 μm (Fig. 3D). Moreover, carbohydrate-degrading specialists were preferentially selected under the larger pore condition. *B. amyloliquefaciens* and *B. subtilis*, both known for high amylase production [43], were substantially enriched in the 150 μm system (Fig. 3F). The normalized stochasticity ratio (NST) values for the 50 μm and 150 μm systems were 76.6% and 67.2%, respectively (Supplementary Fig. S9), indicating that stochastic processes predominated in shaping bacterial community structure.

We further investigated co-occurrence patterns to elucidate the potential ecological interactions among microbial communities across distinct pore-sizes. The microbial network within the 150 μm pore-size exhibited greater complexity and connectivity, as evidenced by a higher number of edges compared to the network in the 50 μm pore-size (Fig. 3G; Supplementary Table S2). Topological features of the network, including average path length and network diameter, were smaller for communities within the 150 μm pore-size compared to those in the 50 μm, although other metrics such as clustering coefficient and modularity remain to be determined (Supplementary Table S3). Moreover, the 150 μm pore-size network exhibited a higher proportion of positive species-level interactions (85.6% vs. 65.2% in the 50 μm, indicative of more cooperative interspecies relationships under larger pore conditions (Fig. 3G). These findings suggest that larger pore-sizes selectively enrich for metabolically beneficial taxa and promote cooperative interactions that support more efficient substrate utilization and metabolite production. A reduction in negative interactions may facilitate the coexistence of functionally complementary taxa and contribute to more stable and efficient ecosystem functioning. Consistent with our findings, studies in soil ecosystems have shown that mitigation of spatial constraints results in higher microbial richness and the emergence of more interconnected and robust co-occurrence network structures [23]. Glucose addition to soil has been shown to strongly enrich specific substrate-utilizing taxa such as *Pseudomonas*, highlighting the link between resource availability and selective microbial proliferation [16].

### Larger pore-size enhances microbial metabolic activity through transcriptional reprogramming

**Figure 4:**
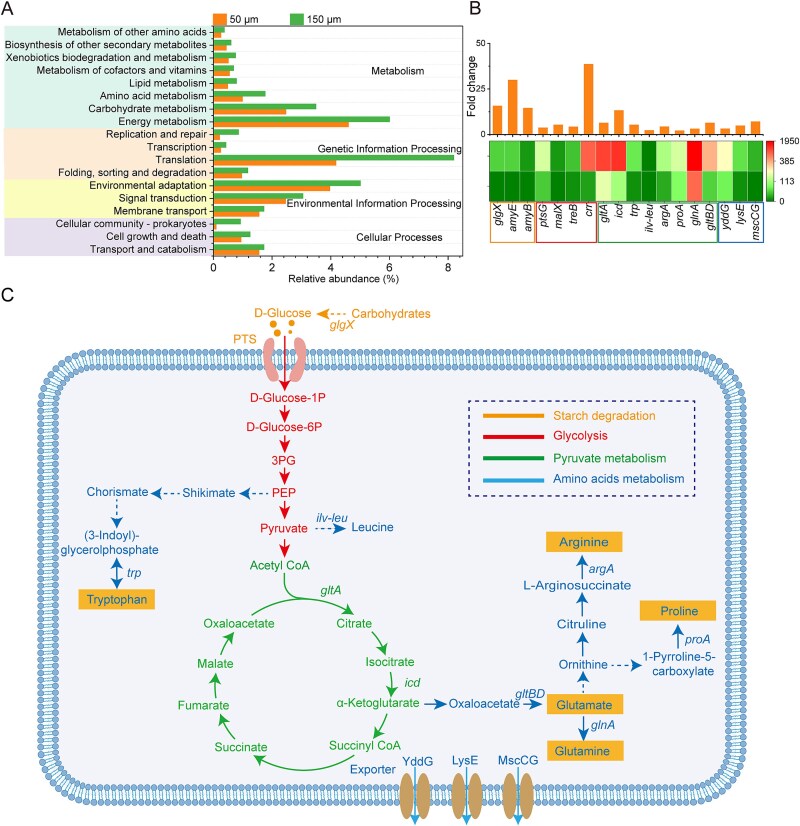
Reconstruction of metabolic networks and abundance of substrate degradation and amino acids metabolism pathways based on the KEGG database annotation. (A). The relative abundances of critical functions based on KEGG level 2 classifications. (B). Abundance and fold change of key genes with different colored in the 50 μm and 150 μm systems corresponding to their respective functions. (C). Metabolic network reconstruction showing reaction directions (arrows) and functional modules (color-coded regions). Annotated genes (above arrows) represent key steps involved in the reaction. Meta-transcriptomics was performed in three replicates.

Microbial metabolic functions are fundamentally governed by gene expression patterns, with transcriptional regulation serving as the primary mechanism linking genetic potential to biochemical activity [44]. Our meta-transcriptomic analysis revealed that pore-size architecture exerts significant influence on the expression of key functional genes, particularly those involved in substrate degradation and amino acid biosynthesis pathways (Fig. 4A). Specifically, increased pore-size had a significant positive impact on the gene functional potential of saccharifying hydrolases involved in carbohydrates degradation. In the 150 μm pore-size, transcript levels of *glgX* (glycogen debranching enzyme), *amyE* (α-amylase), and *amyB* (β-amylase) [45] increased (14.5–29.9-fold), reflecting a strong enhancement in carbohydrate hydrolytic efficiency. Complementing these changes, genes involved in carbohydrate transport were also upregulated in the 150 μm system. (Fig. 4B). Functional genes encoding components of the phosphotransferase system (PTS), particularly the Glc family transporters (*ptsG*, *malX*, *treB*, and *crr*) [46], showed significant increases in expression (3.7–38.6-fold) (Fig. 4B). This signified that the active transport of carbohydrate substrates from extracellular to intracellular cells for subsequent metabolism was significantly enhanced in the larger pore-size. Correspondingly, the carbohydrate metabolism pathways, particularly those related to glucose, exhibited significant enhancements in the 150 μm. We observed coordinated upregulation of the TCA cycle, primarily driven by the dominant genera, as indicated by increased expression of citrate synthase (*gltA*) and isocitrate dehydrogenase (*icd*) [47] (Fig. 4B and C; Supplementary Fig. S10), suggesting larger pore- sizes promoted efficient carbon flux through glycolysis, generating pyruvate which is the key precursor for subsequent amino acids biosynthesis, while simultaneously enhancing ATP production to meet biosynthetic demands.

Microbial communities in the 150 μm pore-size system exhibited significantly enhanced expression of genes encoding key enzymes involved in amino acid biosynthesis compared to the 50 μm system. For example, there was upregulation of the *trp* operon responsible for Trp biosynthesis, the *ilv-leu* operon involved in branched-chain amino acid production, and the *argA* and *proA* genes encoding enzymes for Arg and Pro biosynthesis (2.3 to 5.3-fold). The pore-size dependent transcriptional changes extended to nitrogen metabolism, with the 150 μm system showing higher expression of *glnA* (glutamine synthetase) and *gltBD* (Glu synthase), which are critical for nitrogen assimilation into amino acid backbones. In parallel, genes encoding amino acid efflux transporters were significantly upregulated, including *yddG* (3.2-fold), *lysE* (4.8-fold), and *mscCG* (7.1-fold), suggesting improved amino acid export capacity in the larger pore environment (Fig. 4B and C). Collectively, these transcriptional responses pointed to a pore-size-dependent metabolic reprogramming that enhances both substrate utilization and biosynthetic output. Our findings are consistent with previous study reporting that genes involved in glycolysis/gluconeogenesis, the TCA cycle, and nitrogen metabolism are enriched in soils where glucose was introduced into both small and large pores [16].

### Microbial regulatory adaptations to support enhanced metabolic production under increased pore-size conditions

**Figure 5:**
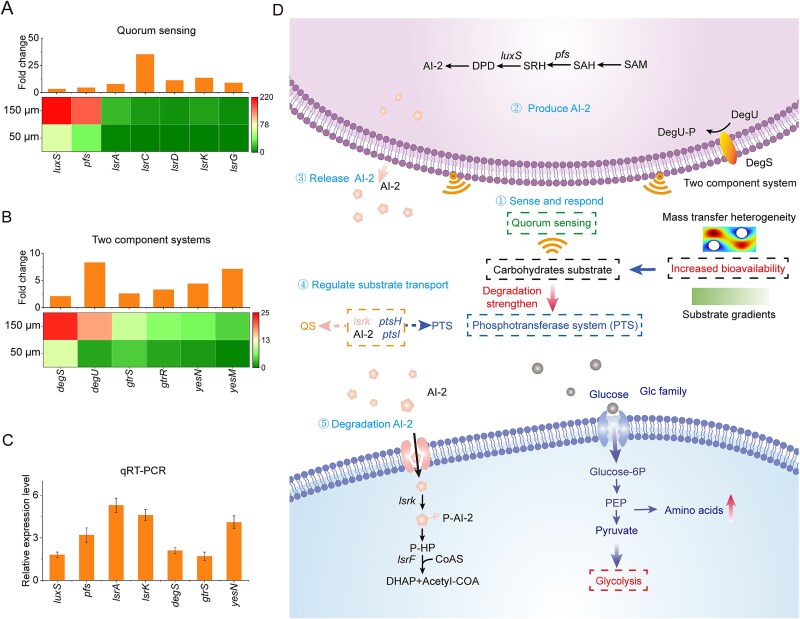
Quorum sensing and two-component regulatory adaptations in response to larger pore-size. (A). The relative abundance of genes encoded by TCSs. (B). The relative abundance of genes encoded by QS systems. (C). The qRT-PCR analysis of representative genes involved in TCSs and QS systems. (D). Schematic representation of microbial substrate transport under modulating spatial constraints. Data are presented as mean ± SD.

Microbial communities employ sophisticated regulatory networks to adapt to dynamic environmental cues, particularly those associated with nutrient availability and metabolic stress [48, 49]. In the context of larger pore-size architecture (150 μm), we observed a substantial shift in the expression of key genes associated with environmental sensing and metabolic coordination, indicative of adaptive regulatory responses that support elevated amino acids production. Meta-transcriptomic analysis revealed an increase in the relative abundance of environmental adaptation-related transcripts in the 150 μm system (Fig. 4A). Among these regulatory systems, QS, particularly the autoinducer-2 (AI-2)-mediated pathway, emerged as a typical mechanism for community-level coordination in response to accelerated substrate degradation [50, 51]. Our results demonstrated that the 150 μm pore system showing the increase in the relative abundance of *luxS* and *pfs* (encoding S-ribosylhomocysteine lyase and adenosylhomocysteine nucleosidase, respectively) compared to the 50 μm system (Fig. 5A). Clearly, the faster metabolic activity observed when carbohydrates served as the primary metabolic substrate facilitated the release and synthesis of AI-2, aligning with previous study that accelerated substrate degradation favored AI-2 secretion. Then, the AI-2-dependent bacteria could sense AI-2 and influence their behavior via the Lsr operon. The encoding genes responsible for AI-2 perception regulation (*lsrA*, *lsrC*, *lsrD*, *lsrK*, and *lsrG*) were increased (6.7–35.1-fold) in the 150 μm than that of the 50 μm. Genes encoding PTS components such as *ptsH* and *ptsI*, which are known to be responsive to AI-2 signaling, also showed significant upregulation (Fig. 5A), highlighting enhanced carbohydrate uptake via the PTS pathway by leveraging AI-2-mediated QS signaling.

Two component systems (TCSs) represent another fundamental microbial regulatory mechanism that maintains cellular homeostasis through adaptive responses to environmental stimuli [52]. Our data demonstrate significant upregulation of the stress-responsive TCS components *degS* (2.1-fold) and *degU* (8.3-fold) which are involved in starch degradation. Additionally, ATP-binding cassette (ABC) transport proteins, particularly *xylG* (4.3-fold) and *xylH* (7.6-fold), which are involved in the sensing, uptake, and utilization of carbohydrates including glucose, were also significantly increased in the 150 μm pore system (Fig. 5B). These transcriptomic patterns were independently validated by quantitative RT-PCR, and the elevated AI-2 concentrations were confirmed by HPLC analysis (Fig. 5C; Supplementary Fig. S11). Collectively, the co-activation of AI-2-dependent QS and TCS-mediated environmental sensing suggests a dual-layered regulatory framework that may enable microbial consortia in the 150 μm system to coordinate substrate acquisition, stress mitigation, and metabolic output, although future validation using mutant strains or chemical inhibitors will be necessary to confirm the causal roles of these signaling pathways (Fig. 5D). Moreover, similar regulatory responses have been observed in anaerobic sludge fermentation, where microbial substrate utilization and expression of metabolic adaptation genes were enhanced during volatile fatty acid promotion [33].

### Pore-size dependent heterogeneity in mass transfer affects substrate availability and microbial function

**Figure 6:**
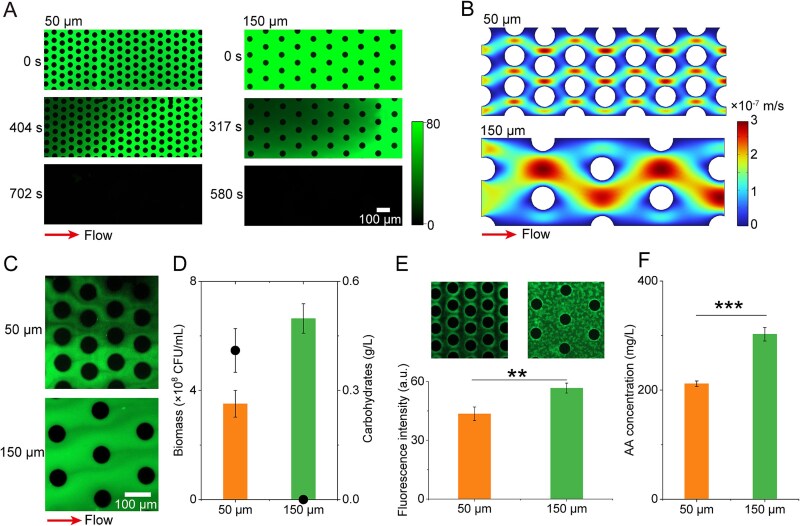
Variations in pore-size alter mass transfer dynamics, impacting substrate distribution and microbial activity. (A). Normalized concentration maps of fluorescein sodium salt over time during displacement by deionized water in 50 μm and 150 μm pore-sizes (*n* = 5). (B). Simulated velocity fields for 50 μm and 150 μm pore-sizes generated using COMSOL Multiphysics. The flow through the pores was modeled under steady-state conditions using the Navier–Stokes equations, solved by the finite element method with linear elements and default stabilization settings in COMSOL. No-slip boundary conditions were applied to all liquid–solid interfaces. (C). Representative snapshots showing concentration gradients of carbohydrates probed with FITC-ConA in porous chips with different pore-sizes. (D). Biomass and carbohydrate content at 2 days postinoculation with *B. Subtilis* Bs01 in each chip type (*n* = 5). (E). Fluorescence intensity of cells expressing *egfp* under the control of the *amyE* promoter. Background fluorescence was measured and subtracted from all images to ensure accurate quantification. (F). Total amino acid concentrations in 50 μm and 150 μm pore-size systems following Bs01 inoculation (*n* = 3). Data are presented as mean ± SD. ^**^P < .01; ^***^P < .001.

Spatial heterogeneity in porous environments critically influences microbial access to nutrients and the establishment of physicochemical gradients, both of which shape microbial community behavior and metabolic activity [3, 24]. Building upon prior research demonstrating that pore architecture regulates solute transport and redox stratification [53], we examined how pore-size affects mass transfer dynamics and subsequent microbial function within microfluidic environments mimicking porous ecosystems. To quantify solute transport behavior, we employed fluorescein sodium as a passive tracer and monitored its displacement by deionized water under constant flow [3]. The residence time of the tracer was significantly longer in the 50 μm pore system (702 s) than in the 150 μm system (580 s), indicating restricted solute mobility in smaller pore environments (Fig. 6A). This prolonged persistence may be attributed to the coupling between restricted flow paths and limited molecular diffusion in the finer matrix. Quantitative image analysis showed that the effective diffusion coefficient (De) in the 50 μm pore-size was reduced by up to 60% compared to the 150 μm pore-size (*P* < .05) (Supplementary Fig. S12). These findings were further supported by simulated velocity fields using COMSOL Multiphysics, which effectively demonstrated the significant impact of pore-scale structural heterogeneity on transport dynamics (Fig. 6B). Physical constraints on diffusion directly influenced substrate gradients within the pore systems. Salek *et al.* (2024) revealed that micromodels with smaller pores have been shown to exhibit earlier breakthrough of deionized water and longer residence time tails for fluorescent tracers [13]. Time-resolved concentration mapping using extract medium labeled with a fluorescently labeled carbohydrate probe (FITC-ConA) revealed pronounced solute gradients within the porous matrices. Smaller pores led to denser packing and narrower pore throats, resulting in restricted diffusion and steeper solute gradients. In contrast, larger pores enabled more homogeneous and sustained substrate availability through enhanced diffusive and convective fluxes, thereby promoting stable and consistent substrate availability (Fig. 6C).

To further verify the impact of pore-scale mass transfer heterogeneity on microbial activity, we isolated an enriched *B. subtilis* strain (Bs01) from the original environmental sample and inoculated it into microfluidic devices. Biomass accumulation was significantly higher in the 150 μm system compared to the 50 μm system (*P* < .01) (Fig. 6D). Correspondingly, carbohydrate degradation rates were faster in the 150 μm pores, indicating improved substrate accessibility and utilization efficiency (Fig. 6D). To probe microbes at the genetic level, we constructed a transcriptional reporter strain harboring *egfp* under the control of the *amyE* promoter, which encodes α-amylase, a key enzyme in polysaccharide hydrolysis. Fluorescence microscopy revealed a 1.3-fold higher expression of the reporter in the 150 μm environment, consistent with upregulated transcriptional activity of hydrolytic enzymes (Fig. 6E). This was further validated by quantitative enzymatic assays, which demonstrated higher extracellular enzyme production under the same conditions (Supplementary Fig. S13). Moreover, the concentration of secreted amino acids was also 43.13% higher in the 150 μm system, suggesting enhanced biosynthetic capacity (Fig. 6F). While this synthetic reporter system enables high-resolution tracking of gene expression in a model organism, its scalability to natural complex communities is limited by genetic inaccessibility and interspecies interactions.

Collectively, our results provide strong evidence that larger pore dimensions enhance microbial growth and metabolic activity by improving mass transfer, nutrient access, and spatial organization. These findings are consistent with recent studies demonstrating the dynamic interplay between hydrodynamics and biofilm formation in porous media, which collectively govern nutrient accessibility and microbial colonization patterns [5]. Pores within the 100–150 μm range have been particularly associated with elevated enzymatic activities and an expanded microbial spatial footprint in soil, contributing to enhanced carbon storage capacity [14]. Additionally, hydrologically driven nutrient redistribution, such as flow-stimulated cellulase activity, has been shown to modulate organic matter decomposition and nutrient fluxes in aquatic sediments [54]. While our microfluidic system simplifies certain aspects of natural porous environments, it offers a powerful and highly controlled platform to isolate and investigate pore-scale effects that are difficult to disentangle in more complex systems. The fermentation-derived inoculum employed in this study is functionally diverse and broadly representative of carbon-rich ecosystems. The application of steady, laminar flow captures key features of mass transfer dynamics, providing mechanistic insight into microbial responses to hydrodynamic conditions, even though it does not fully mimic the heterogeneous and transient flows characteristic of natural settings. Beyond pore-size, other key physicochemical factors, such as pore geometry and flow velocity [11, 21], also play pivotal roles in shaping microbial development within porous ecosystems. Further investigations building on this platform can systematically explore the impact of additional physical parameters, including shear stress, flow variability, and partial saturation, to better approximate natural porous ecosystems.

## Conclusion

Our study demonstrates that pore-scale physical structure is a fundamental driver of microbial community assembly and function in porous ecosystems. By integrating a microfluidic platform with multi-omics approaches, we show that larger pore-sizes alleviate mass transfer limitations, leading to enhanced substrate degradation, metabolic activity, and functional gene expression. These spatial configurations selectively enrich metabolically specialized taxa and promote more complex community interactions, emphasizing the critical role of microscale heterogeneity in shaping both the ecological and functional architecture of microbial communities. These findings advance our mechanistic understanding of how physical constraints within structured environments govern microbial processes, with broad implications for managing microbial functions in natural and engineered porous systems, including the design of structured bioreactors, the optimization of soil microenvironments, and the targeted modulation of host-associated microbiomes.

## Supplementary Material

Supplemental_Material_wraf205

## Data Availability

Raw metagenomic and metatranscriptomic data have been deposited in the National Center for Biotechnology Information (NCBI) Sequence Read Archive (SRA) under the accession number PRJNA1262537 and PRJNA1263651.
