# Supplementary material for: Pore-scale mass transfer heterogeneity shapes nutrient accessibility and functional assembly in porous microbial ecosystems
Source: ISME J. 2025 Sep 12;19(1):wraf205. doi: 10.1093/ismejo/wraf205 (PMC12499919; doi:10.1093/ismejo/wraf205)
Supplement: Supplemental_Material_wraf205 [file supplemental_material_wraf205.docx]

**Pore-Scale Mass Transfer Heterogeneity Shapes Nutrient Accessibility and Functional Assembly in Porous Microbial Ecosystems**

Liming Wu ^1^, Daixiu Bao ^1^, Hui Liao ^2^, Meiyu Yan ^1^, Yitong Ge ^1^, Zinuan Han ^1^, Xiaole Xia ^1, 2*^

^1^ College of Food Science and Engineering, Tianjin University of Science and Technology, Tianjin, 300457, P. R. China.

^2^ The Key Laboratory of Industrial Biotechnology, Ministry of Education, School of Biotechnology, Jiangnan University, Wuxi, 214122, P. R. China.

***Corresponding author**

Xiaole Xia: xiaxiaole@tust.edu.cn

College of Food Science and Engineering, Tianjin University of Science and Technology, No. 9, 13th Avenue, Tianjin Economic-Technological Development Area, Tianjin, 300457, P. R. China.

**Supplementary Text S1. Microﬂuidic devices and experiments**

Microfluidic chambers were engineered to mimic porous environmental architectures using a matrix of micropillars (50 μm diameter and height). Two distinct microfluidic designs were fabricated, differing in interpillar spacing to produce pore-sizes of 50 μm and 150 μm, respectively. Microstructures were created via deep reactive ion etching (DRIE) following standard photolithographic protocols, with development performed in an AZ400K:H₂O (1:4) solution. The etched silicon wafers served as master molds for replicating polydimethylsiloxane (PDMS) microchannels. The patterned PDMS layers were bonded to glass slides after plasma treatment to create enclosed microfluidic devices.

*Daqu* extract medium was prepared from the same *Daqu* sample using a modified method. Brieﬂy, 500 g of *Daqu* was subjected to two rounds of extraction with 1.3 L of 80% methanol to yield the supernatant, which was subsequently filtered and lyophilized. The lyophilized sample was then reconstituted in 200 mL water and passed through a 0.22 μm ﬁlter. The ﬁltrate was designated as the *Daqu* extract solution. 1 L of extract medium consisted of 0.2 L extract solution, 0.23 g KH_2_PO_4_, 0.23 g K_2_HPO_4_, 0.23 g MgSO_4_·7H_2_O, 0.33 g NH_4_NO_3_, 0.25 g NaHCO_3_, 1 mL of a vitamin stock solution (thiamine hydrochloride, 0.5 g/L; riboﬂavin, 0.5 g/L; niacin, 0.5 g/L; pyridoxine HCl, 0.5 g/L; inositol, 0.5 g/L; calcium pantothenate, 0.5 g/L; β-aminobenzoic acid, 0.5 g/L; biotin, 0.25 g/L), 2 mL of a selenite-tungstate solution (NaOH, 0.5 g/L; Na_2_SeO_3_·5H_2_O, 3 mg/L; Na_2_WO_4_·2H_2_O, 4 mg/L) and 2 mL of the trace element solution (HCl, 2.8 g/L; FeCl_2_·4H_2_O, 1.5 g/L; ZnCl_2_, 70 mg/L; MnCl_2_·4H_2_O, 100 mg/L; H_3_BO_3_, 6 mg/L; CoCl_2_·6H_2_O, 190 mg/L; CuCl_2_·2H_2_O, 2 mg/L; NiCl_2_·6H_2_O, 24 mg/L; Na_2_MoO_4_·2H_2_O, 36 mg/L). The pH of the medium was adjusted to 7.0 using either HCl or NaOH.

**Supplementary Text S2. Exometabolomic analysis**

To assess the influence of pore-size on microbial metabolic activity, 100 μL of effluent at 3 d was collected from the microfluidic chip outlet for metabolomic profiling. Samples were filtered through a 0.2-μm membrane to remove bacterial cells, and the filtrate was mixed with 400 μL of pre-chilled 80% methanol, vortexed, and centrifuged at 15,000 × g for 20 minutes at 4 °C. The resulting supernatant was vacuum-dried and reconstituted in 100 μL of acetonitrile/water (1:1, v/v), followed by centrifugation (14,000 × g, 15 min, 4 °C) prior to liquid chromatography-mass spectrometry (LC-MS) analysis. Metabolomic profiling was performed using an ultra-high performance liquid chromatography system (Vanquish UHPLC) coupled to a Q Exactive HF-X hybrid quadrupole-Orbitrap mass spectrometer (Thermo Fisher Scientific). Data acquisition was carried out in both positive and negative electrospray ionization (ESI) modes, with a full-scan mass range of m/z 100-1500.

Raw data were converted to mzXML format using ProteoWizard and processed with XCMS package. Only level 2 identified metabolites (putatively annotated) were used for downstream analysis. Differentially abundant metabolites (DAMs) were identified via orthogonal partial least squares discriminant analysis (OPLS-DA) combined with t-tests (VIP > 1, *P* < 0.05). Pathway enrichment of DAMs was performed using the Kyoto Encyclopedia of Genes and Genomes (KEGG) database via the MetaboAnalyst 5.0.


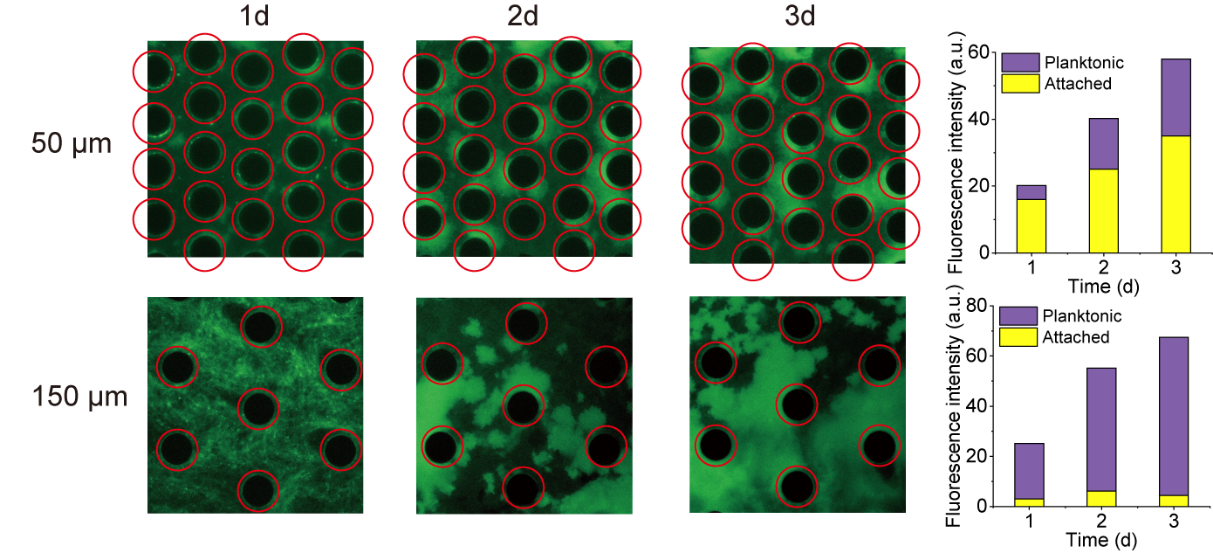


**Supplementary Fig. S1. Ratios of attached and planktonic cells in 50 μm and 150 μm pore-sized chips during 3 days of incubation.** Cells within the red circle represent attached cells. The assay was conducted in three independent replicates.


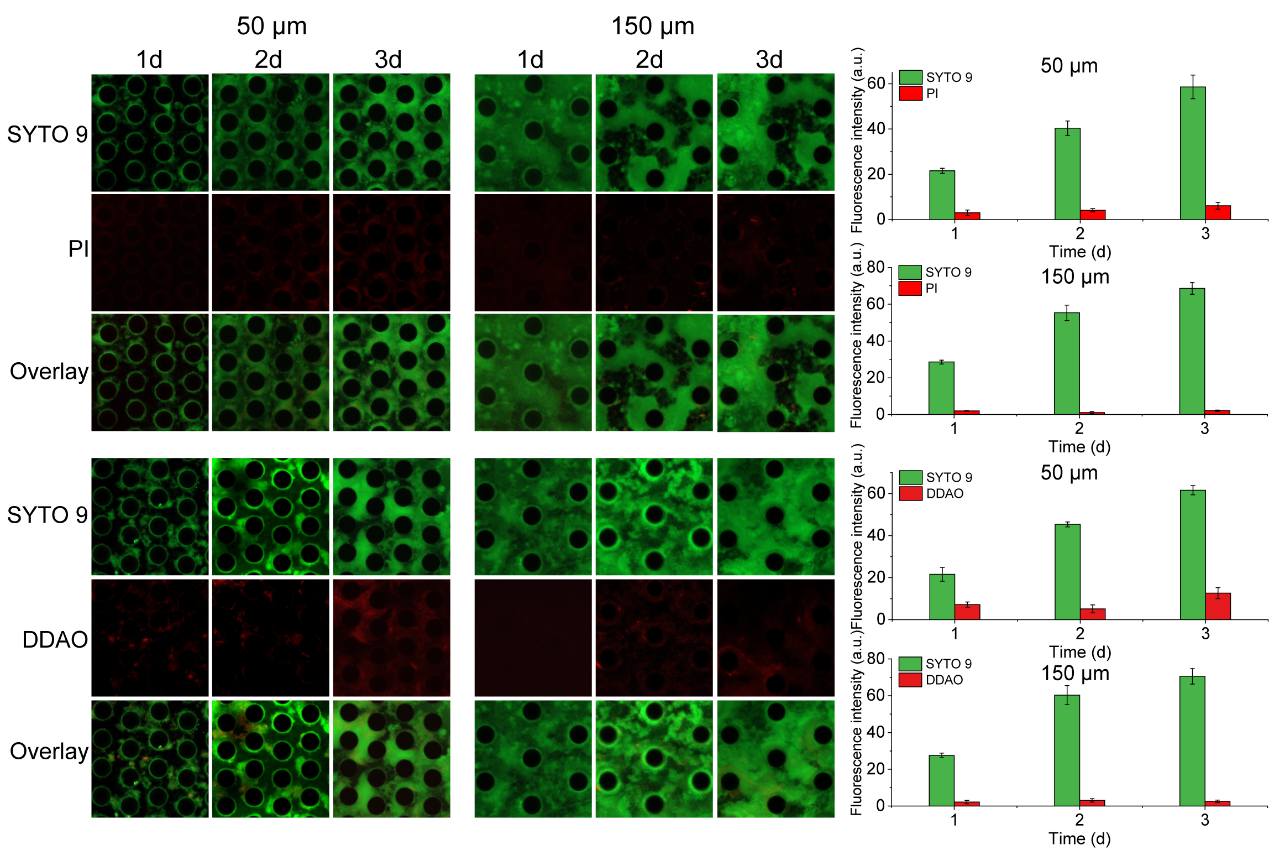


**Supplementary Fig. S2. Time-lapse imaging of microbial community architecture in a porous environment.** Cells were stained with SYTO 9 (green) to visualize total biomass. Propidium iodide (PI) was used to identify dead cells, and DDAO (7-hydroxy-9H-(1,3-dichloro-9,9-dimethylacridin-2-one)) was applied to selectively label extracellular DNA. The assay was conducted in three independent replicates.


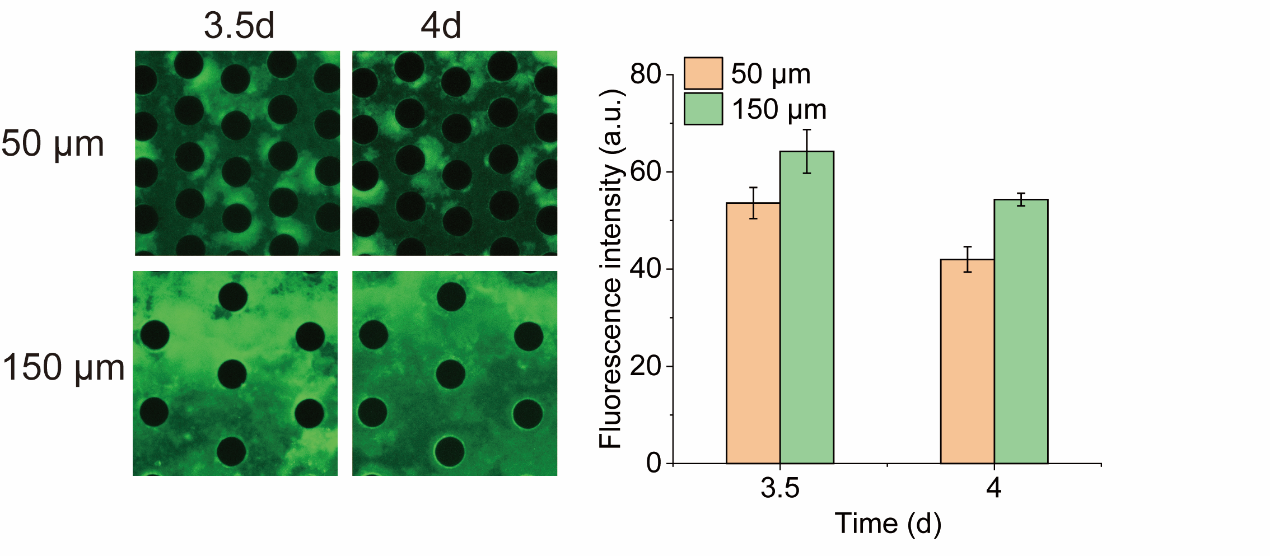


**Supplementary Fig. S3. Time-lapse imaging analysis and fluorescence intensity of the microbial community in 50 μm and 150 μm pore-size microfluidic chips at day 3.5 and day 4.** The fluorescence intensity at day 3.5 showed no significant difference compared to that at day 3 (*P* > 0.05), while it decreased at day 4, suggesting that both microfluidic systems reached the stationary growth phase after 3 days of incubation. The assay was conducted in three independent replicates.


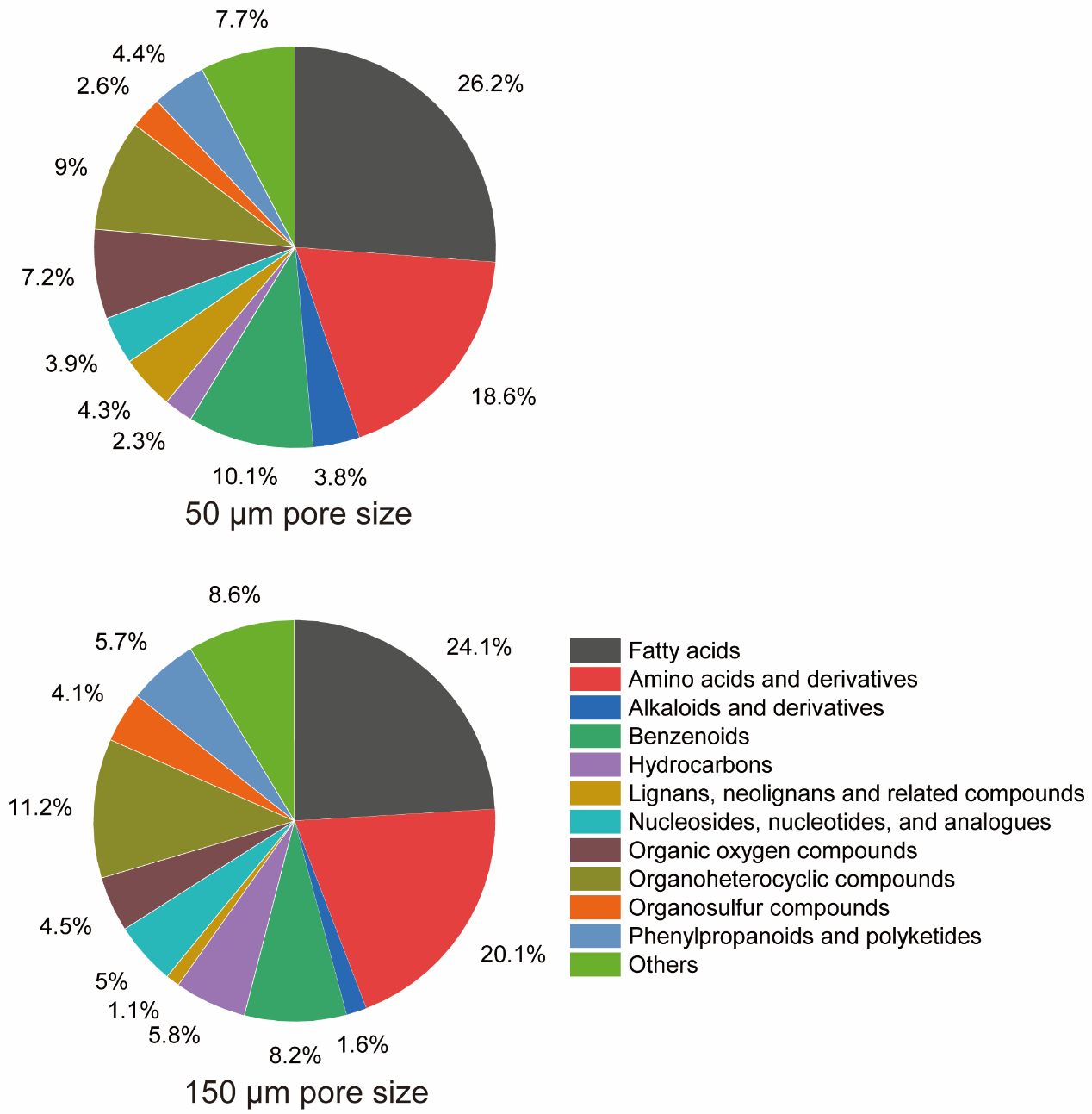


**Supplementary Fig. S4. Metabolites identified by untargeted metabolomics analysis in 150 μm and 50 μm pore-sizes.** A total of 1417 and 1542 metabolites were detected in the 50 μm and 150 μm pore-sizes, respectively. Fatty acids (26.2% and 24.1%) and amino acids (AAs) along with their derivatives (18.6% and 20.1%) were the main constituents. Metabolomics was performed in three replicates.


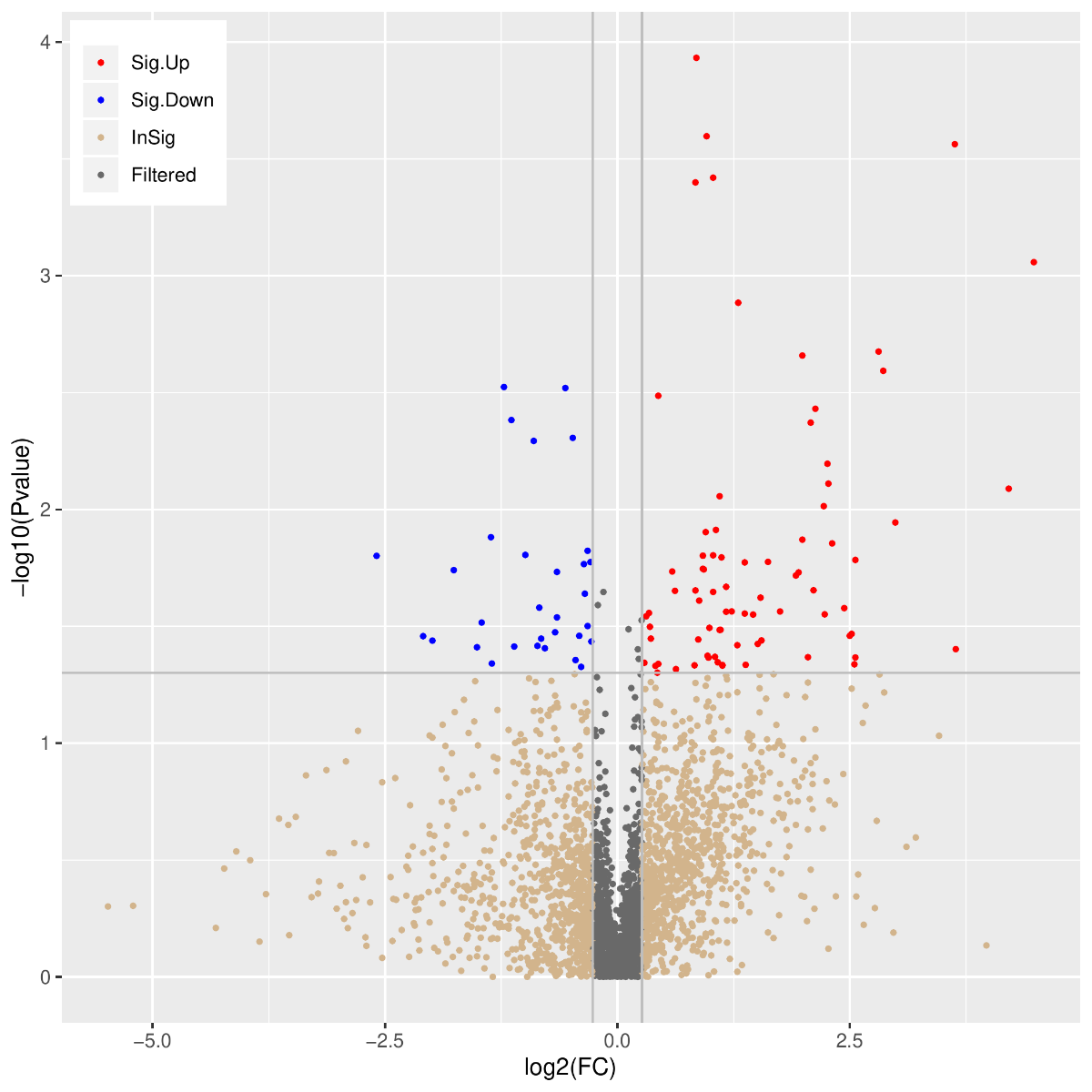


**Supplementary Fig. S5. Volcano plot analysis of differential metabolites between** **50 μm and 150 μm pore-sizes.** A total of 109 metabolites exhibited significant differences. In the 150 μm pore-size group, 78 metabolites were upregulated and 31 were downregulated compared to the 50 μm group. The color of the dots represents the metabolite categories. Red: Significantly upregulated metabolites (meeting the threshold)**.** Blue: Significantly downregulated metabolites (meeting the threshold). Gray: Metabolites that did not meet the fold-change threshold**.** Tan: Metabolites that met the fold-change threshold but not the *P*-value threshold.

**
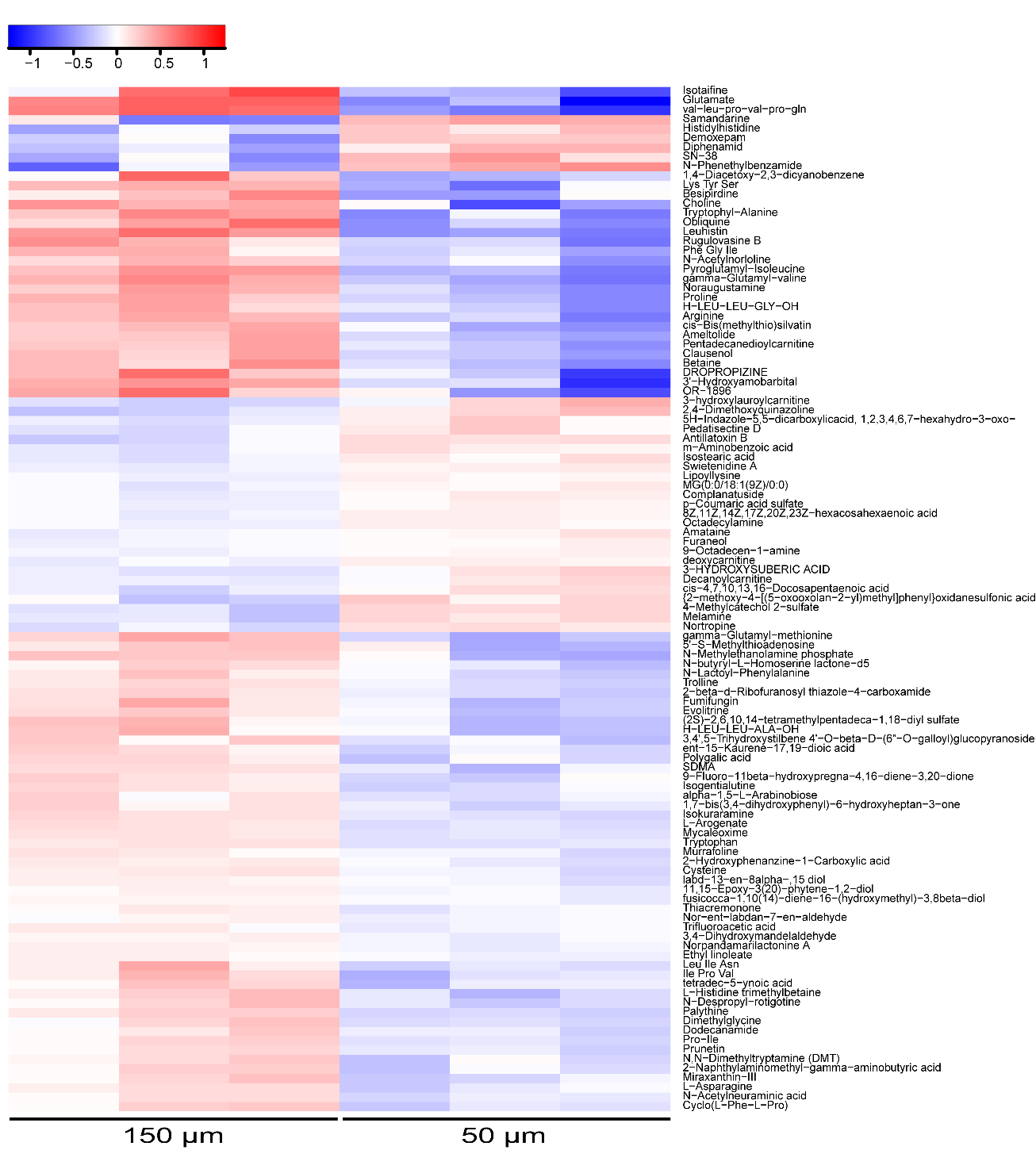
**

**Supplementary Fig. S6. The different metabolites exhibiting VIP scores >1.0 using OPLS-DA analysis between the 150 μm and 50 μm groups.** 78 metabolites were upregulated and 31 were downregulated in the 150 μm group relative to the 50 μm group.

**
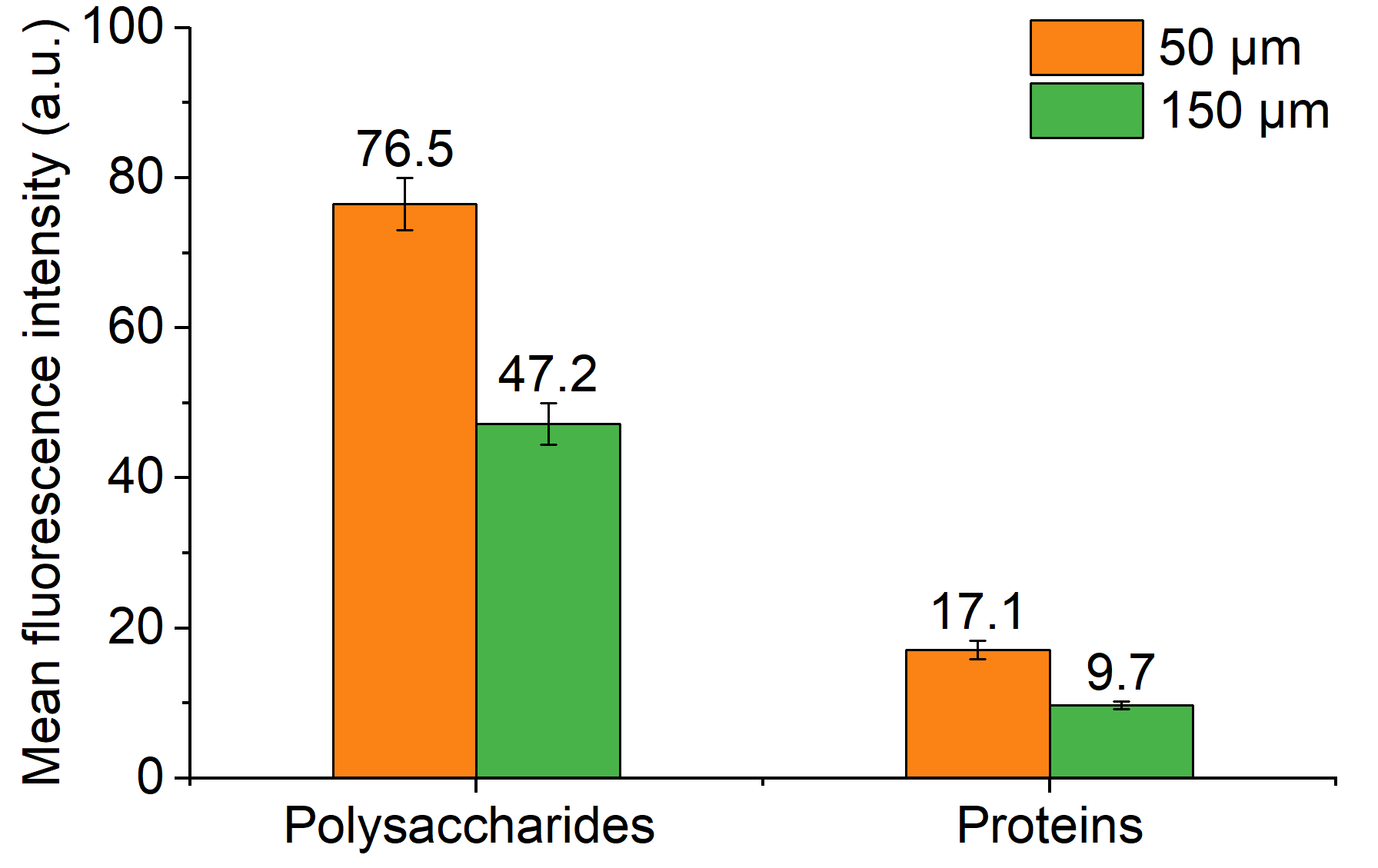
**

**Supplementary Fig. S7. The mean fluorescence intensity of polysaccharides and proteins at 2 d incubation in the 50 μm and 150 μm pore-sizes chips (n=5).**

**
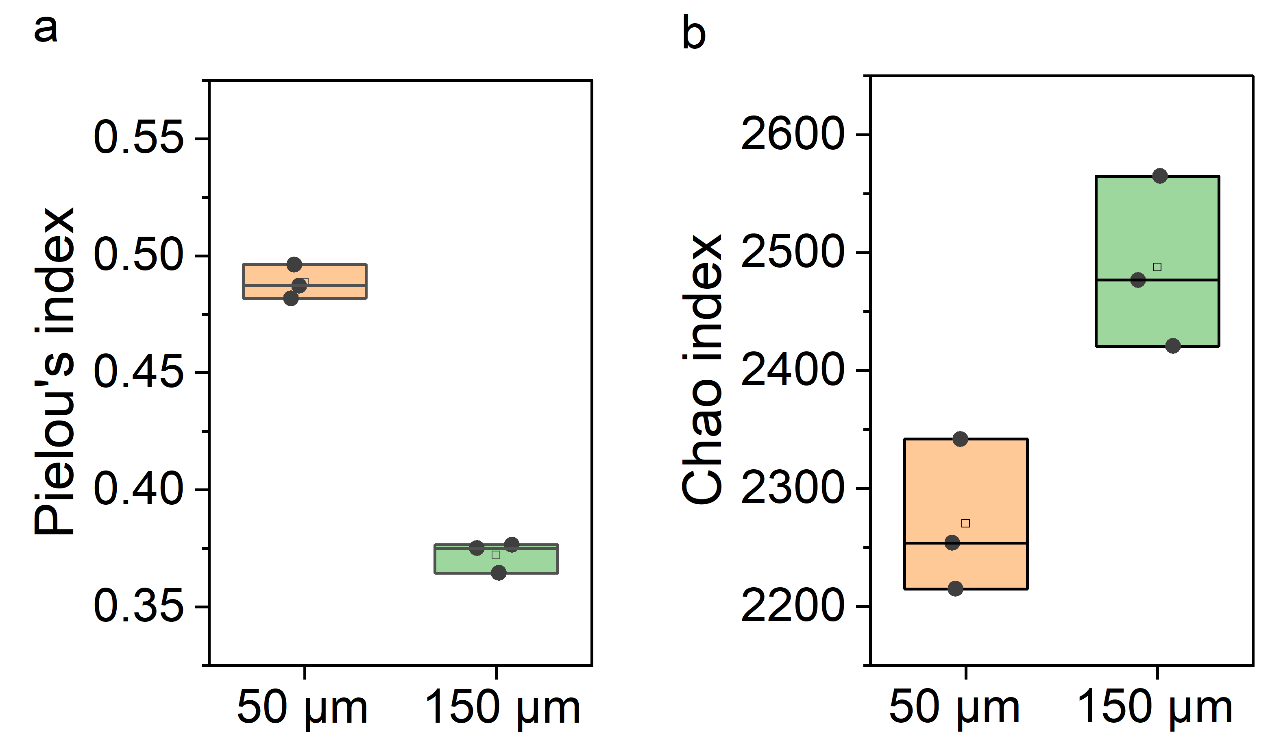
**

**Supplementary Fig. S8. Taxonomic diversities of bacterial communities across 50 μm and 150 μm pore-sizes.** a: Pielou's index represents community evenness. b: The Chao index represents taxonomic richness. Metagenomics was performed in three replicates.


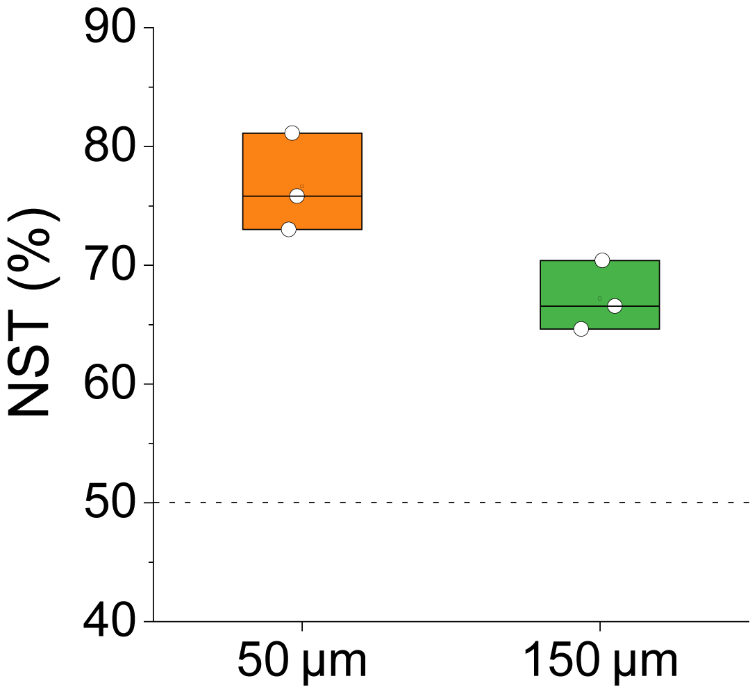


**Supplementary Fig. S9. The normalized stochasticity ratio under different pore-size conditions.**


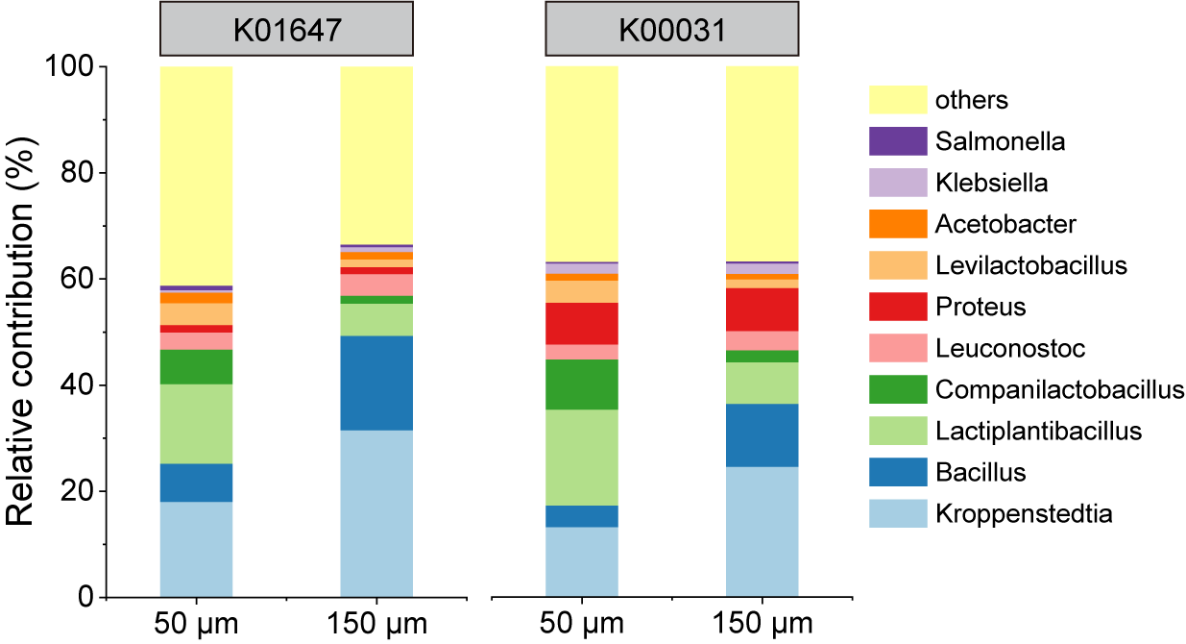


**Supplementary Fig. S10. The relative genus-level contributions of K01647 (*gltA*) and K0031 (*icd*) in the 50 μm and 150 μm systems.**


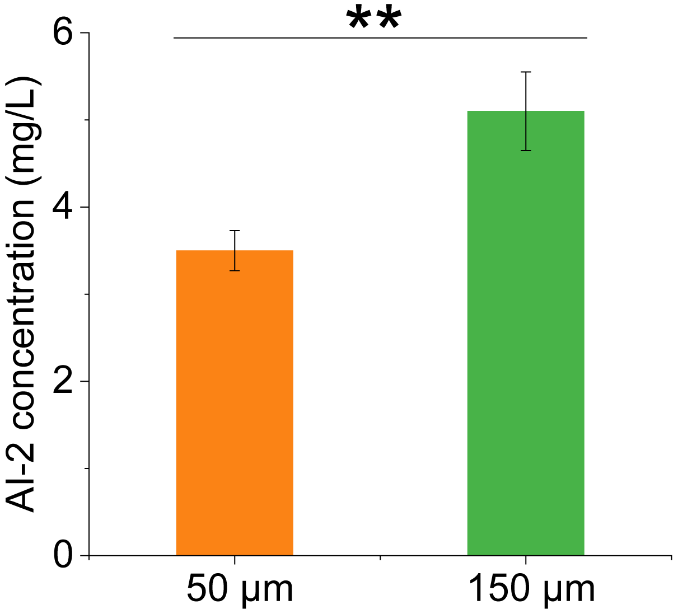


**Supplementary Fig. S11. The AI-2 concentrations in the 50 μm and 150 μm pore-size systems at day 3.** The assay was conducted in three independent replicates.


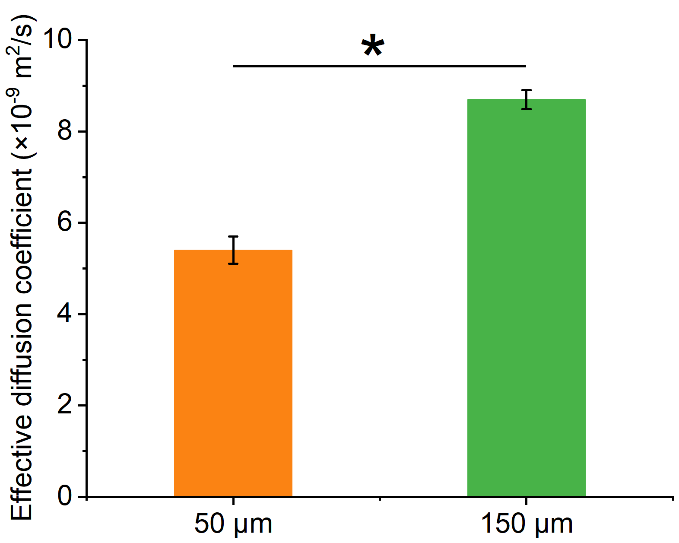


**Supplementary Fig. S12. The effective diffusion coefficients for pore-sizes of 50 μm and 150 μm.** The coefficients were calculated using the equation provided in the study by de Anna et al. (2021).

de Anna P, Pahlavan AA, Yawata Y *et al.* Chemotaxis under flow disorder shapes microbial dispersion in porous media. *Nat Phys* 2021;**17**:68-73


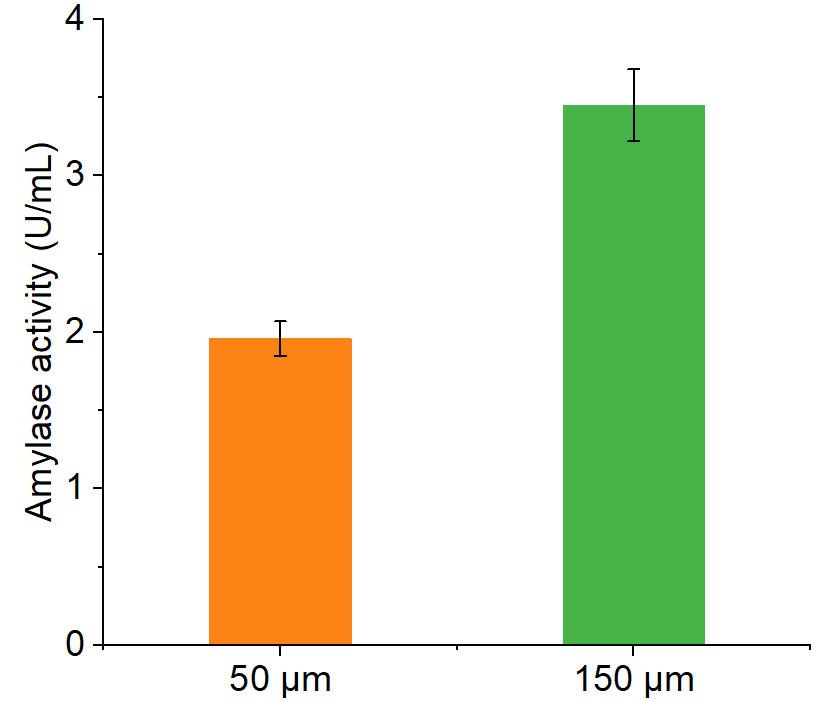


**Supplementary Fig. S13. Amylase activity associated with carbohydrate hydrolysis in 50 μm and 150 μm pore-size systems, as measured using an amylase assay kit (n=5).**

**Supplementary Table S1. Primers used in this study.**

| Name | Sequence (5´-3´) |
| --- | --- |
| 338F | ACTCCTACGGGAGGCAGCA |
| 806R | GGACTACHVGGGTWTCTAAT |
| 16S-F | CTAACCCGCCGCCCTTAT |
| 16S-R | CTGCGAATGATTTCCAAC |
| LuxS-F | AAACGGTGAGCAGTCAATCA |
| LuxS-R | AAGGTCCTAAGGGTGACAAGA |
| pts-F | TAAGATCTCATGAATAAAAAGGATTATAAT |
| pts-R | TACTCGAGATCACCACTTATTTTCAC |
| lsrA-F | TGCTCGTGAACCTGAAGTGC |
| lsrA-R | CGATAGCCAGCAGCAATACC |
| lsrK-F | AGCAGCGTTAACGAGTTGGA |
| lsrK-R | TCATCGTCGTAGCCATCAGG |
| degS-F | CGCTGGTGAAATCGTTCAGT |
| degS-R | TCTTCCAGCGATAACGACCT |
| gtrS-F | GGTGGTGCTGAAGTCTACGA |
| gtrS-R | CGACAGGATGTCGAAGTAGC |
| yesN-F | CGGCTGCTGTTGTTATTGGT |
| yesN-R | ACGCAGATGTCATAGCCGTT |
| amyE-F | ATGTTTGCAAAACGATTCA |
| amyE-R | TTCTTCTCCCTTACCCATTCAATGGGGAAGAGAACCG |
| egfp-F | CGGTTCTCTTCCCCATTGAATGGGTAAGGGAGAAGAA |
| egfp-R | TTACATGTTTGTTCATCATTTAGGCGGGCTGCCCCGG |
| lut-F | CCGGGGCAGCCCGCCTAAATGATGAACAAACATGTAA |
| lut-R | TTAGTTGACTTTTTGTTCT |

**Supplementary Table S2. Microbial community abundance at the family level in 50 μm and 150 μm pore-sizes.**

| **Family level** | **50 μm** | **150 μm** |
| --- | --- | --- |
| f__Thermoactinomycetaceae | 0.302635 | 0.434784 |
| f__Lactobacillaceae | 0.417953 | 0.186873 |
| f__Bacillaceae | 0.08907 | 0.209069 |
| f__Streptomycetaceae | 0.035644 | 0.024825 |
| f__Morganellaceae | 0.029596 | 0.02922 |
| f__Enterobacteriaceae | 0.018311 | 0.018995 |
| f__Acetobacteraceae | 0.019748 | 0.014576 |
| f__Paenibacillaceae | 0.009489 | 0.010193 |
| f__Staphylococcaceae | 0.007018 | 0.011352 |
| f__Pseudonocardiaceae | 0.011312 | 0.000905 |
| f__Streptococcaceae | 0.006213 | 0.006135 |
| f__Moraxellaceae | 0.005776 | 0.003903 |
| f__Shewanellaceae | 0.006171 | 0.003356 |
| f__Planococcaceae | 0.000533 | 0.006009 |
| f__Clostridiaceae | 0.001879 | 0.002386 |
| f__Candidatus_Scalinduaceae | 0.002278 | 0.001518 |
| f__Nitrobacteraceae | 0.001816 | 0.001742 |
| f__Enterococcaceae | 0.00211 | 0.001353 |
| f__Sporolactobacillaceae | 0.001303 | 0.00189 |
| f__Pseudomonadaceae | 0.001385 | 0.001357 |
| others | 0.029761 | 0.029559 |

**Supplementary Table S3. Network-level topological features.** Co-occurrence networks of the first 25 species were constructed using Spearman’s rank correlation, highlighting both positive and negative correlations (Spearman’s r > 0.5, *P* < 0.05). Their structural properties were characterized by calculating topological parameters with the NetworkAnalyzer plugin

| Parameter | 50 μm | 150 μm |
| --- | --- | --- |
| Edges | 112 | 139 |
| Network diameter | 13 | 8 |
| Average path length | 4.712 | 2.980 |
| Average degree | 4.21 | 2.543 |
